# Health of mothers of children with a life-limiting condition: a comparative cohort study

**DOI:** 10.1136/archdischild-2020-320655

**Published:** 2021-03-02

**Authors:** Lorna K Fraser, Fliss EM Murtagh, Jan Aldridge, Trevor Sheldon, Simon Gilbody, Catherine Hewitt

**Affiliations:** 1 Health Sciences, University of York, York, UK; 2 Hull York Medical School, University of Hull, Hull, Kingston upon Hull, UK; 3 Clinical Psychology, Leeds Teaching Hospitals NHS Trust, Leeds, UK; 4 School of Medicine, Queen Mary University of London, London, UK

**Keywords:** palliative care, epidemiology

## Abstract

**Objective:**

This study aimed to quantify the incidence rates of common mental and physical health conditions in mothers of children with a life-limiting condition.

**Methods:**

Comparative national longitudinal cohort study using linked primary and secondary care data from the Clinical Practice Research Datalink in England. Maternal–child dyads were identified in these data. Maternal physical and mental health outcomes were identified in the primary and secondary care datasets using previously developed diagnostic coding frameworks. Incidence rates of the outcomes were modelled using Poisson regression, adjusting for deprivation, ethnicity and age and accounting for time at risk.

**Results:**

A total of 35 683 mothers; 8950 had a child with a life-limiting condition, 8868 had a child with a chronic condition and 17 865 had a child with no long-term condition.

The adjusted incidence rates of all of the physical and mental health conditions were significantly higher in the mothers of children with a life-limiting condition when compared with those mothers with a child with no long-term condition (eg, depression: incidence rate ratio (IRR) 1.21, 95% CI 1.13 to 1.30; cardiovascular disease: IRR 1.73, 95% CI 1.27 to 2.36; death in mothers: IRR 1.59, 95% CI 1.16 to 2.18).

**Conclusion:**

This study clearly demonstrates the higher incidence rates of common and serious physical and mental health problems and death in mothers of children with a life-limiting condition. Further research is required to understand how best to support these mothers, but healthcare providers should consider how they can target this population to provide preventative and treatment services.

**What is already known on this topic?**

- There are growing numbers of children with life-limiting conditions in which the mothers provide healthcare 24 hours, 7 days a week.
- There is evidence of an increased risk of mortality among mothers whose infant has died or has a significant congenital anomaly.
- Most healthcare services focus on individual patients and not the whole family, thus ignoring the needs of parents.

**What this study adds?**

- Mothers of children with a life-limiting condition have significantly higher incidence of depression, anxiety and serious mental illness than other mothers.
- They also have significantly higher incidence of cardiovascular disease, hypertension and mortality.
- Much of this morbidity may be preventable.

There are more than 86 000 children living in England with conditions^1^ which will either ultimately shorten their life (eg, Leigh’s disease) or conditions for which treatment may be available but may fail (eg, cancer).^2^ The defining feature of children with a life-limiting or life-threatening condition is that these children are at risk of premature death, and dying in childhood or early adulthood may be expected. Now, these children are living longer in part due to the more aggressive management of complications^3^ and the increasing use of medical technologies (eg, home ventilation).^4^

It is often expected that parents of these children, predominately the mother,^5^ become healthcare providers as well as parents, 24 hours a day 7 days a week. The health of these mothers is important, both in terms of caring for their child but also in their own right to health and well-being. Most healthcare services focus on individual patients and not the whole family, therefore ignoring the needs of parents.

The lack of studies quantifying the mental health of mothers of children with a life-limiting condition has been highlighted by the National Institute for Health and Care Excellence.^6^ Although studies show that mothers of children with special needs^7^ or specific disabilities^8 9^ have shown higher levels of parental distress or emotional problems than parents of healthy children, these studies do not address the specific needs of those with life-limiting conditions or the added burden that their parents face, knowing their child is likely to die.

There is evidence of an increased risk of mortality among mothers whose infant has died or has a significant congenital anomaly.^10^ ^11^ However, there is little evidence about the physical health of mothers of children with life-limiting conditions. Two cross-sectional studies in mothers of children with disabilities found higher prevalence of self-reported physical conditions compared with mothers of healthy children (eg, back pain, 35.2% vs 26.7%, and hypertension, 24.7% vs 19.1%).^9 12^

Quantifying and understanding the physical and mental health of these mothers is vital before any effective interventions can be designed, targeted or tested.^6^ Therefore, this study aims to quantify the incidence of commonly occurring mental and physical health conditions in mothers of children with a life-limiting condition using a nationally representative longitudinal healthcare dataset.

## Methods

This observational comparative cohort study was conducted in accordance with a protocol and reported according to the Strengthening the Reporting of Observational Studies in Epidemiology-RECORD guidelines.^13^

### Data sources

The study used an anonymised extract of data from the Clinical Practice Research Datalink (CPRD) GOLD dataset, which contains longitudinal primary care records from a representative sample of general practitioner (GP) practices across the UK (covering approximately 8.5% of the UK population)^14^ linked to records from secondary care data (Hospital Episodes Statistics (HES) and the Mental Health Minimum Dataset (MHMDS))^15 16^ and Office for National Statistics (ONS) death certificate data. The datasets were linked using deterministic methods by CPRD using NHS number, sex, date of birth and postcode,^16^ and mothers were linked to their children using the CPRD mother–baby link algorithm, which is based on pregnancy records.^15^

The CPRD GOLD dataset^14^ contains information on consultations, prescriptions and referrals. HES contains information about clinical diagnosis and procedures, and patient information including age, sex and ethnicity, for all inpatient stays.^17^ MHMDS contains information on individuals who have received specialist secondary mental healthcare, including outpatient, inpatient and community care.^18^

### Cohort identification

The cohort was identified by the CPRD team via the disease group of the children (see online supplemental material). The identification of life limiting and chronic disease in the children was undertaken using previously developed Read code frameworks (primary care) or International Classification of Diseases code frameworks (secondary care) for life-limiting^19 20^ and chronic conditions^21^ in children.

10.1136/archdischild-2020-320655.supp1 Supplementary data

The index children (life-limiting conditions) were included if they were eligible for HES linkage (ie, resident in England) and where the mother had at least 1 year of registration in the CPRD dataset, between 1 April 2007 and 31 December 2017. These eligible children were then matched to children with chronic conditions (1:1) or no long-term conditions (1:2) on year of birth, sex and geographical region. All primary and secondary care for the child–mother dyads were extracted.

### Outcomes

The health outcomes for mothers were identified by the authors using Read coded data in the CPRD GOLD dataset (clinical interaction data including symptoms, diagnoses, referrals and prescriptions) or International Classification of Diseases, 10th Revision, diagnostic codes in the secondary healthcare data. These outcomes are common health conditions seen in primary care and could be plausibly linked to the physical or psychological pressure of having a child with a chronic or life-limiting condition. The code lists for each outcome were identified using previously published studies (online supplemental material).

#### Mental health outcomes

- Anxiety.^22^
- Depression.^22^
- Serious mental illness (schizophrenia and bipolar disorder).^23^
- Referral to secondary mental health services (present in the MHMDS).

#### Physical health outcomes

- Back pain^24^
- Obesity^25 26^
- Hypertension^24^
- Cardiovascular disease (CVD).^22^
- Type 2 diabetes mellitus^25 26^
- Death, via the linkage to the ONS death registration data.

Time at risk was calculated separately for each outcome of interest and from the point of child’s diagnosis to the recording of the outcome of interest or end date of the mother. Incidence rates were calculated per person years at risk for each outcome.

Mothers who had a diagnosis of an outcome of interest prior to the record of diagnoses in their child were excluded from the analyses only for that outcome—this enabled us to exclude diagnoses in the mothers that occurred prior to their child’s diagnosis.

### Other variables of interest

The age of the mother was calculated as the age at their entry to this study.

The deprivation category, a measure of socioeconomic status (split into five groups using the Index of Multiple Deprivation 2010),^27^ was provided as linked data based on the most recent known address of the individual.

The ethnic group (black African, black Caribbean, black Other, Chinese, Bangladeshi, Indian, Pakistani, other Asian, white, mixed or other^28^) was recorded in the linked HES data, where an individual had more than one ethnic group, provided it was set by CPRD to the most commonly recorded value, excluding unknown. Due to the small number in some of these ethnic groups, categories were collapsed into six groups; white, South Asian, black, Chinese, mixed and other.

Smoking status was using the Read code list available for current smoking status.^22^

### Statistical methods

Crude incidence rates of the physical and mental health conditions were calculated in each group of mothers by dividing the number of cases in each group by the person-time at risk in each group.

Multivariable Poisson models were built for each outcome of interest and included maternal age, ethnicity, deprivation status, number of GP consultations and the matching variables (child birth year, child sex and region) to compare the incidence rates between the groups of mothers using incidence rate ratios (IRRs) and accounting for time at risk. Confounding variables were retained if they improved the model fit (via Bayesian Information Criterion).

Analyses were undertaken using STATA V.15.^29^

### Patient and public involvement

The views of parents and carers of children with a life-limiting condition informed the development of this study, including refining the research question.

## Results

The cohort for analyses contained 35 683 mothers, of whom 8950 had a child with a life-limiting condition; 8868 had a child with a chronic condition; and 17 865 had a child with no long-term condition (table 1).

**Table 1:** Participant characteristics

	Child has a life-limiting condition		Child has a chronic condition		Child has no long-term condition		Total	
	n	%		%		%		%
Total mothers	8950		8868		17 865		35 683	
Mothers’ mean age (years) (SD)	34.0 (7.7)		33.8 (7.3)		34.1 (7.2)		34.0 (7.4)	
Min–max	15–64		15–62		15–62		15–64	
Deprivation category								
1 (least deprived)	1853	20.7	2037	23.0	4596	25.7	8486	23.8
2	1826	20.4	1749	19.7	3597	20.1	7172	20.1
3	1732	19.4	1685	19.0	3365	18.8	6782	19.0
4	1827	20.4	1753	19.8	3319	18.6	6899	19.3
5 (most deprived)	1706	19.1	1642	18.5	2979	16.7	6327	17.7
Missing	6	0.1	2	0.0	9	0.1	17	0.0
Ethnic group								
White	7272	81.3	7341	82.8	14 578	81.6	29 191	81.8
South Asian	584	6.5	520	5.9	940	5.3	2044	5.7
Black	323	3.6	310	3.5	524	2.9	1157	3.2
Chinese	42	0.5	29	0.3	94	0.5	165	0.5
Mixed	90	1.0	80	0.9	165	0.9	335	0.9
Other	156	1.7	133	1.5	310	1.7	599	1.7
Unknown	483	5.4	455	5.1	1254	7.0	2192	6.1
Number of GP consultations in analyses period								
Median	20		29		22		23	
Q1, Q3	9, 39		15, 51		11, 39		11, 42	
Min–max	1–391		1–451		1–451		1–451	
Region								
North East	223	2.5	220	2.5	439	2.5	882	2.5
North West	1446	16.2	1439	16.2	2888	16.2	5773	16.2
Yorkshire and Humber	257	2.9	248	2.8	511	2.9	1016	2.8
East Midlands	249	2.8	240	2.7	495	2.8	984	2.8
West Midlands	971	10.8	968	10.9	1940	10.9	3879	12.8
East of England	1145	12.8	1141	12.9	2288	12.8	4574	12.8
South West	1157	12.9	1140	12.9	2311	12.9	4608	12.9
South Central	1118	12.5	1104	12.4	2229	12.5	4451	12.5
London	1317	14.7	1308	14.7	2634	14.7	5259	14.7
South East Coast	1067	11.9	1060	12.0	2130	11.9	4257	11.9
Length of follow-up (years)								
Mean (SD)	6.7 (3.4)		7.8 (3.1)		7.5 (3.2)		7.3 (3.2)	
Min–max	1.1–12.1		1.0–12.1		1.0–12.1		1.0–12.1	
Current smoker	2098	23.4	2228	25.1	4133	23.1	8459	23.7

GP, general practitioner.

There were few missing data apart from ethnic group (6% unknown ethnicity). Unknown ethnic group was retained as a category for analyses (table 1).

Mothers of children with a life-limiting condition on average visited the GP less frequently (median=20) than mothers of children with a chronic condition (median=29, table 1).

The numbers of mothers removed from each incidence analyses as they were diagnosed prior to their child’s diagnoses are as follows:

- Depression 10 558.
- Anxiety 5862.
- Serious mental illness 165.
- Referral to secondary mental health services 820.
- Hypertension 1308.
- CVD 76.
- Type 2 diabetes 332.
- Back pain 12 193.

The crude incidence rates of depression, anxiety, serious mental illness and referral to secondary mental health services are significantly higher in the mothers of children with a life-limiting or chronic condition when compared with mothers whose children have no long-term condition (table 2).

**Table 2:** Crude incidence rates of physical and mental health conditions in mothers by diagnostic group of the child

	Child has a life-limiting condition			Child has a chronic condition			Child has no long-term condition		
	Incident cases (n)	Incidence per 10 000 person years	95% CIs	Incident cases (n)	Incidence per 10 000 person years	95% CIs	Incident cases (n)	Incidence per 10 000 person years	95% CIs
Mental health outcomes									
Depression	1196	341	322 to 361	1343	340	322 to 359	2350	268	257 to 279
Anxiety	917	201	188 to 214	1104	212	200 to 225	1816	168	160 to 176
Serious mental illness	60	10.1	7.8 to 13	55	8	6.2 to 10.4	55	5.5	4.3 to 6.8
MHMDS	712	46.2	40.7 to 52.3	647	37.5	33 to 42.6	1022	26.8	24.1 to 29.8
Physical health outcomes									
Obesity	693	128	119 to 138	711	115	107 to 124	1126	91.1	85.9 to 96.6
Cardiovascular disease	80	13.4	10.8 to 16.7	59	8.6	6.7 to 11.1	86	6.4	5.2 to 7.9
Hypertension	470	84.3	77 to 92.2	512	79.3	72.8 to 86.6	725	57.1	53.1 to 61.4
Type 2 diabetes	168	28.7	24.7 to 33.4	180	26.6	23 to 30.7	271	20.3	18.1 to 22.1
Back pain	1316	402	381 to 424	1641	471	449 to 495	2835	364	351 to 377
Death	68	11.4	9.0 to 14.4	41	6.0	4.4 to 8.1	91	6.8	5.5 to 8.3

IRR, incidence rate ratio; MHMDS, Mental Health Minimum Dataset.

The crude incidence rates of obesity, hypertension, type 2 diabetes and back pain are significantly higher in the mothers of children with a life-limiting or chronic condition when compared with mothers whose children have no long-term condition; for example, for depression, crude incidence rates were 341 (95% CI 322 to 361), 340 (95% CI 322 to 359) and 268 (95% CI 257 to 259) per 10 000 person years, respectively. The crude incidence rates of CVD are significantly higher in mothers of children with a life-limiting condition (13.4 per 10 000 person years, 95% CI 10.8 to 16.7), but not in those of a child with a chronic condition (8.6 per 10 000 person years, 95% CI 6.7 to 11.1) when compared with mothers whose children have no long-term condition (6.4 per 10 000 person years, 95% CI 5.2 to 7.9).

The crude rate of death (11.4 per 10 000 person years, 95% CI 9.0 to 14.4) was significantly higher in mothers of children with a life-limiting condition, but not in those of a child with a chronic condition (6.0 per 10 000 person years, 95% CI 4.4 to 8.1) when compared with mothers whose children have no long-term condition (6.8 per 10 000 person years, 95% CI 5.5 to 8.3; table 2). The univariate models are available in the online supplemental material.

**Figure 1:**
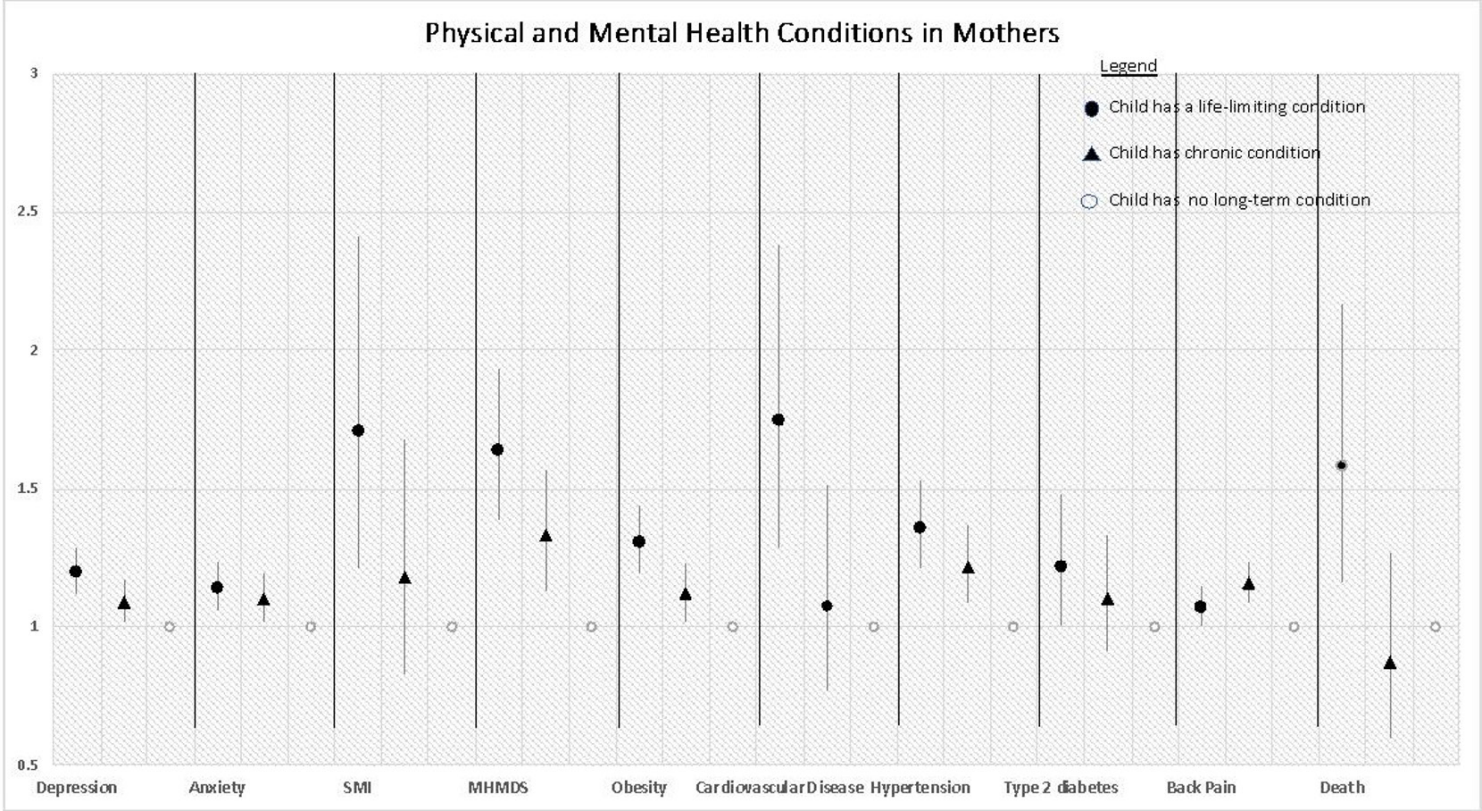
Physical and mental health conditions in mothers; adjusted incidence rate ratios (models adjusted for age of mother, index of multiple deprivation, ethnic group, number of general practitioner consults; smoking status was also included in the models for cardiovascular disease and hypertension).

There is significantly higher incidence of all mental health outcomes in mothers of children with a life-limiting condition when compared with mothers whose children have no long-term condition (eg, depression IRR 1.21, 95% CI 1.13 to 1.30) in the adjusted analyses (figure 1 and table 3). For mothers whose child has a chronic condition the incidence of depression, anxiety and referral to secondary mental health services are significantly higher than for mothers whose children have no long-term condition, but their incidence of serious mental illness was not significantly different (IRR 1.17, 95% CI 0.82 to 1.67).

**Table 3:** Multivariable models for maternal mental health outcomes

	Anxiety	n=29 392	Depression	n=24 754	Serious mental illness	n=35 036	Referral to secondary mental health services	n=32 842
	IRR	95% CI	IRR	95% CI	IRR	95% CI	IRR	95% CI
Child has no long-term condition	REF		REF		REF		REF	
Child has a life-limiting condition	1.16	1.07 to 1.25	1.21	1.13 to 1.30	1.66	1.17 to 2.34	1.61	1.37 to 1.90
Child has a chronic condition	1.11	1.03 to 1.19	1.09	1.02 to 1.17	1.17	0.82 to 1.67	1.17	0.98 to 1.38
Mothers’ age	0.97	0.97 to 0.98	0.97	0.96 to 0.97	0.94	0.92 to 0.96	0.95	0.94 to 0.96
Deprivation category								
1 (least deprived)	REF		REF		REF		REF	
2	0.99	0.89 to 1.10	1.06	0.97 to 1.15	1.28	0.76 to 2.15	1.65	1.29 to 2.14
3	1.13	1.02 to 1.25	1.12	1.03 to 1.23	1.25	0.74 to 2.11	1.88	1.46 to 2.42
4	1.15	1.04 to 1.27	1.23	1.13 to 1.35	1.69	1.03 to 2.76	2.00	1.56 to 2.57
5 (most deprived)	1.16	1.04 to 1.29	1.37	1.24 to 1.50	1.68	1.00 to 2.81	2.09	1.61 to 2.70
Ethnic group								
White	REF		REF		REF		REF	
South Asian	0.52	0.44 to 0.62	0.44	0.38 to 0.51	0.32	0.12 to 0.86	0.62	0.43 to 0.89
Black	0.43	0.32 to 0.56	0.54	0.45 to 0.66	0.78	0.28 to 2.18	0.51	0.30 to 0.86
Chinese	0.76	0.44 to 1.30	0.35	0.19 to 0.65	0.00	0	0.00	0.00
Mixed	0.96	0.69 to 1.33	0.91	0.68 to 1.22	1.94	0.61 to 6.14	0.62	0.26 to 1.49
Other	0.66	0.48 to 0.89	0.57	0.44 to 0.74	1.05	0.33 to 3.33	0.94	0.53 to 1.68
Missing	0.63	0.53 to 0.76	0.70	0.60 to 0.81	0.23	0.06 to 0.93	0.51	0.32 to 0.81
Number of GP consultations	1.01	1.01 to 1.01	1.01	1.01 to 1.01	1.01	1.01 to 1.01	1.01	1.01 to 1.01
Region								
North East	2.11	1.73 to 2.57	1.61	1.34 to 1.94	0.91	0.30 *to 2.73*	0.73	0.44 to 1.24
North West	1.43	1.27 to 1.62	1.29	1.16 to 1.43	1.56	0.89 *to 2.72*	0.53	0.39 to 0.72
Yorkshire and Humber	1.04	0.83 to 1.30	0.93	1.16 to 1.43	0.21	0.03 *to 1.58*	1.05	0.67 to 1.65
East Midlands	2.09	1.68 to 2.61	2.20	1.81 to 2.68	3.37	1.45 to 7.87	1.62	1.00 to 2.64
West Midlands	1.24	1.08 to 1.42	1.19	1.06 to 1.33	1.02	0.54 to 1.91	1.04	0.79 to 1.37
East of England	1.05	0.91 to 1.20	1.00	0.89 to 1.13	1.04	0.54 to 2.01	0.61	0.44 to 0.84
South West	1.37	1.20 to 1.55	1.19	1.07 to 1.34	0.80	0.42 to 1.53	1.87	1.47 to 2.38
South Central	1.13	0.99 to 1.29	1.23	1.09 to 1.38	1.12	0.59 to 2.12	0.29	0.19 to 0.44
London	REF							
South East Coast	1.03	0.90 to 1.18	1.07	0.96 to 1.20	1.09	0.58 to 2.03	1.16	0.89 to 1.51
Child sex								
Male	REF							
Female	0.97	0.91 to 1.04	1.02	0.96 to 1.08	0.94	0.70 to 1.26	1.05	0.91 to 1.21
Baby birth year	1.01	1.01 to 1.02	1.03	1.02 to 1.03	0.96	0.93 to 0.99	0.99	0.98 to 1.01

GP, general practitioner; IRR, incidence rate ratio; REF, reference.

For all the physical health outcomes in mothers (figure 1 and table 4), the incidence rates are significantly higher in mothers of children with a life-limiting condition when compared with mothers whose children have no long-term condition (eg, CVD IRR 1.73, 95% CI 1.27 to 2.36). For mothers whose child has a chronic condition, the incidence of obesity, hypertension and back pain are significantly higher than for mothers whose children have no long-term condition, but their incidence of type 2 diabetes (IRR 1.09, 95% CI 0.90 to 1.32) and CVD (IRR 1.06, 95% CI 0.76 to 1.49) was not significantly different.

**Table 4:** Multivariable models for maternal physical health outcomes

	Obesity	n=32 675	Cardiovascular disease	n=35 122	Hypertension	n=33 904	Type 2 diabetes	n=34 869	Back pain	n=23 111
	IRR	95% CI	IRR	95% CI	IRR	95% CI	IRR	95% CI	IRR	95% CI
Child has no long-term condition	REF		REF		REF		REF		REF	
Child has a life-limiting condition	1.32	1.20 to 1.45	1.73	1.27 to 2.36	1.35	1.20 to 1.52	1.22	1.01 to 1.48	1.08	1.01 to 1.15
Child has a chronic condition	1.12	1.03 to 1.23	1.06	0.76 to 1.49	1.21	1.08 to 1.36	1.09	0.90 to 1.32	1.16	1.09 to 1.23
Mothers’ age	0.98	0.97 to 0.99	1.12	1.09 to 1.14	1.07	1.06 to 1.08	1.07	1.05 to 1.08	0.99	0.99 to 0.99
Deprivation category										
1 (least deprived)	REF		REF		REF		REF		REF	
2	1.67	1.44 to 1.92	1.32	0.80 to 2.19	1.09	0.93 to 1.27	1.02	0.76 to 1.37	1.11	1.02 to 1.20
3	1.89	1.64 to 2.18	2.06	1.29 to 3.30	1.38	1.18 to 1.61	1.67	1.27 to 2.18	1.15	1.06 to 1.25
4	2.27	1.97 to 2.61	3.25	2.08 to 5.07	1.66	1.43 to 1.93	2.06	1.59 to 2.67	1.22	1.12 to 1.32
5 (most deprived)	2.62	2.27 to 3.03	3.54	2.21 to 5.67	1.69	1.44 to 1.99	2.51	1.92 to 3.29	1.30	1.19 to 1.42
Ethnic group										
White	REF		REF		REF		REF		REF	
South Asian	1.08	0.92 to 1.26	1.40	0.83 to 2.36	1.47	1.19 to 1.79	3.32	2.62 to 4.20	1.28	1.15 to 1.42
Black	1.28	1.05 to 1.57	0.94	0.42 to 2.08	2.50	2.00 to 3.13	1.65	1.11 to 2.45	1.26	1.10 to 1.45
Chinese	0.10	0.01 to 0.70	0.00	0.00	1.27	0.60 to 2.68	0.55	0.08 to 3.95	0.62	0.38 to 1.00
Mixed	0.79	0.49 to 1.25	0.00	0.00	1.70	1.05 to 2.75	0.84	0.27 to 2.61	1.11	0.85 to 1.44
Other	0.77	0.54 to 1.09	1.38	0.51 to 3.79	1.10	0.73 to 1.66	1.44	0.79 to 2.64	1.08	0.88 to 1.32
Missing	0.53	0.42 to 0.68	0.18	0.07 to 0.50	0.89	0.73 to 1.08	0.53	0.35 to 0.81	0.75	0.66 to 0.85
Number of GP consultations	1.01	1.01 to 1.01	1.01	1.01 to 1.01	1.01	1.01 to 1.01	1.01	1.01 to 1.01	1.01	1.01 to 1.01
Smoking			1.26	0.95 to 1.67	1.12	1.01 to 1.23				
Region										
North East	1.21	0.93 to 1.57	1.46	0.53 to 4.07	1.21	0.85 to 1.73	1.30	0.78 to 2.19	1.11	0.93 to 1.34
North West	1.03	0.88 to 1.19	1.12	0.30 to 4.18	1.25	1.04 to 1.51	1.12	0.84 to 1.51	1.10	1.00 to 1.21
Yorkshire and Humber	0.98	0.74 to 1.29	2.92	0.88 to 9.76	1.10	0.78 to 1.54	1.79	1.13 to 2.85	1.00	0.84 to 1.19
East Midlands	2.31	1.82 to 2.93	1.16	0.40 to 3.33	2.05	1.49 to 2.83	2.39	1.46 to 3.93	1.36	1.12 to 1.66
West Midlands	1.28	1.09 to 1.49	1.94	0.68 to 5.49	1.16	0.95 to 1.41	1.03	0.75 to 1.41	1.01	0.91 to 1.13
East of England	1.07	0.91 to 1.27	1.12	0.39 to 3.22	1.38	1.14 to 1.67	1.09	0.79 to 1.50	1.08	0.98 to 1.20
South West	1.24	1.06 to 1.44	1.12	0.38 to 3.32	1.02	0.83 to 1.25	1.07	0.77 to 1.47	0.96	0.87 to 1.07
South Central	1.06	0.89 to 1.25	0.92	0.31 to 2.70	1.15	0.94 to 1.41	1.08	0.78 to 1.50	1.06	0.96 to 1.17
London	REF									
South East Coast	0.87	0.74 to 1.03	1.29	0.44 to 3.79	1.23	1.01 to 1.50	0.89	0.62 to 1.26	0.98	0.89 to 1.08
Child sex										
Male										
Female	1.01	0.94 to 1.09	0.88	0.68 to 1.16	0.96	0.87 to 1.06	0.88	0.75 to 1.04	0.91	0.86 to 0.96
Baby birth year	1.01	1.00 to 1.02	1.00	0.98 to 1.03	0.99	0.97 to 1.00	0.99	0.98 to 1.01	1.01	1.00 to 1.02

GP, general practitioner; IRR, incidence rate ratio; REF, reference.

The adjusted incidence rates of death in mothers of children with a life-limiting condition was higher (IRR 1.59, 95% CI 1.16 to 2.18) than those in mothers whose child had no long-term condition (figure 1).

## Discussion

This population-based study has shown that the incidence rates of both common mental and physical health conditions are higher in mothers of children with a life-limiting condition when compared with mothers whose child has no long-term health condition. However, these mothers visited their GP practices less frequently. The risk of death was also more than 50% higher in this population of mothers. Much of this excess morbidity may be preventable through proactive healthcare incorporating both primary and secondary prevention initiatives.

Previous studies assessing the health outcomes of mothers have either been in specific groups of children with intellectual or broader disabilities and have focused on the mental health outcomes.^8 30–34^ The current findings are consistent with a recent meta-analysis that highlighted the increased risk of depressive symptoms and poorer general health of mothers of children with developmental disabilities^34^ and with previous studies of the health of mothers with children with physical disabilities.^30 35^

Many published studies have not differentiated between mothers of children with life-limiting or other chronic conditions.^34 36^ This study differentiates between these groups to address the additional layer of complexity within these mother’s lives in that they are aware that their child will die prematurely^37^ and also enables comparison between the groups to assess the dose–response element of the relationship with the outcomes. A recent cross-sectional study of parents of children being cared for by a palliative care service estimated that nearly half of these parents showed signs of clinically elevated stress, depression or anxiety.^38^

The finding of higher risk of death in this population of mothers is consistent with other published data^10 11^ on the impact of early child death on mothers’ risk of mortality. However, this study includes a group of children with broader age and range of life-limiting diagnoses.^11^ The higher incidence rates of CVD, type 2 diabetes and hypertension in the current study are important risk factors for morbidity and mortality, but these may be amenable to primary or secondary preventative strategies.

While these findings highlight higher incidence rates of physical and mental health conditions, it cannot identify how these mothers could be better supported. Some research supports the use of peer support services to maintain the health and well-being in parents of children with disabilities,^39^ but to date, none have accounted for the additional pressure of being told that your child may die.^40^

These mothers will have many more contacts with paediatric healthcare providers than with their own healthcare provider, and there may be a role of paediatric providers in providing support or signposting to appropriate services. Family centred care is an approach that has highlighted the importance of the family unit when providing health services to children with chronic conditions or disabilities,^41^ but the implementation of this model of care has been limited.^42^ Further research should focus on the most feasible ways to support health needs of this population of mothers.

### Strengths and weaknesses of the study

This was a longitudinal study which used a nationally representative sample of primary and secondary healthcare data.^14^ This allowed the comprehensive identification of the child’s disease status and maternal outcomes of interest. Causality cannot be fully established using an observational study design, but we have demonstrated the temporality of the relationship between exposure and outcome and a dose–response relationship with key health outcomes using as robust a study design as possible.

This study is reliant on the quality of diagnostic coding within the datasets. It is difficult to assess severity or prognoses due to heterogeneity of some conditions and variation in coding practice among GPs. We have no evidence that these coding practices would differ between the groups of mothers. Although we used data on age and smoking, we were missing information on some key confounders, including family history of CVD, nutrition and alcohol intake. Causes of death data were not available.

This study focused on mothers due to the mothers usually, but not exclusively, being the main carers for these children.^5^ It is also not currently possible reliably to identify father–child dyads within the CPRD data.

## Conclusion

This study clearly demonstrates the higher incidence rates of physical and mental health in mothers of children with a life-limiting condition. Further research is required to understand how best to support these mothers, but healthcare providers should consider how they could provide preventative and treatment services for this population.

## Data Availability

Data may be obtained from a third party and are not publicly available. the clinical codes used for this study are provided as supplementary material. The patient level data cannot be shared but can be accessed via the Clinical Practice Research Datalink.
