# Supplementary material for: Health of mothers of children with a life-limiting condition: a comparative cohort study
Source: Arch Dis Child. 2021 Mar 2;106(10):987–93. doi: 10.1136/archdischild-2020-320655 (PMC8461446; doi:10.1136/archdischild-2020-320655)
Supplement: Supplementary data [file archdischild-2020-320655supp001.pdf]

Supplementary Material

Table 1 Cohort identification processes

Groups	Inclusion criteria	Exclusion Criteria
1. Life-limiting or life-threatening condition	• Children from the source population who have prevalent diagnosis event records for life-limiting conditions, either in the Clinical or Referral files in CPRD GOLD based on the Read codes detailed in Supplementary material, or in HES APC based on the ICD-10 codes in Supplementary material.<br>• Children have above events before the end of the study period (31/12/2017).<br>• Children have above events within their UTS follow-up period.<br>• Children are aged 18 years or less on the diagnosis event date.	None
2. Chronic condition that is not life-limiting	One matching control will be provided for each case in Group 1. The controls will comprise of patients from the source population who fulfil the following criteria:<br>• Children from the source population who have diagnosis event records for other chronic conditions in the Clinical or Referral files in CPRD GOLD based on the Read codes detailed in Supplementary material, or in HES APC based on the ICD-10 codes in Supplementary material.<br>• Children have above events before the end of the study period of (31/12/2017).<br>• Children have above events within their UTS follow-up period.<br>• Children are aged 18 years or less on the diagnosis date.	•Children with a diagnosis on or before 31/12/2017 of life-limiting conditions, either in the Clinical or Referral files in CPRD GOLD based on the Read codes detailed in Appendix 1, or in HES APC based on the ICD-10 codes in Appendix 2.<br>• Children who have deregistered from the CPRD on or before 01/04/2007<br>• Children who are siblings of children in the case population (Group 1), based on having a link to the same mother in the Mother Baby Link
3. No long term conditions	Up to two matching controls will be provided for each case in Group 1. The controls will comprise of patients from the source population who fulfil the following criteria:	• Children with a diagnosis of life-limiting conditions on or before 31/12/2017, either in the Clinical or Referral files in CPRD GOLD based on the Read codes detailed in

	• Children from the source population who have at least one day of registration during follow-up start and end.	Appendix 1, or in HES APC based on the ICD-10 codes in Appendix 2, will be excluded<br>• Children with a diagnosis of other chronic conditions on or before 31/12/2017, in the Clinical or Referral files in CPRD GOLD based on the Read codes detailed in Appendix 3, or in HES APC based on the ICD-10 codes in Appendix 4, will be excluded.<br>• Children who have deregistered from the CPRD on or before 01/04/2007<br>• Children who are siblings of children in the case population (Group 1), based on having a link to the same mother in the Mother Baby Link

Cohort definition in CPRD GOLD:

Source Population based on CPRD GOLD:

- 17,029,241 patients are acceptable
- 8,047,360 patients are eligible for linkage to HES, ONS Mortality data and Patient-level Index of Multiple Deprivation data linkage
- 8,047,284 patients are male/female
- 5,408,168 patients have at least one day of UTS follow-up during the study period
- 617,174 patients are included in the Mother Baby link as babies
- 613,259 patients are linked to mothers with eligibility for linkage to HES, ONS Mortality data and Patient-level Index of Multiple Deprivation data linkage
- 565,580 patients are linked to mothers with at least 1 year of UTS follow-up within the study period of 01/04/2007 to 31/12/2017

Case definition (Group 1):

- 11,116 patients have diagnosis event records for life-limiting conditions, either in the Clinical or Referral files in CPRD GOLD based on the Read codes detailed in Appendix 1, or in HES APC based on the ICD-10 codes in Appendix 2.
- 10,598 patients have above events before the end of the study period (31/12/2017).
- 9318 patients have above events before the end of their UTS follow-up period

- 8950 patients are aged 18 years or less on the diagnosis event date

8950 patients meet all criteria.

Of these patients, all are eligible for linkage to ONS Death registration data, Mental Health Services Data Set and patient-level IMD.

8673 patients were identified as mothers of cases in Group 1 in the CPRD Mother Baby Link.

Of these mothers, all are eligible for linkage to ONS Death registration data, Mental Health Services Data Set, and patient-level IMD.

Eligible Control group (Group 2)

From the source population in CPRD GOLD:

- 160,831 patients have diagnosis event records for chronic conditions that are not life-limiting in the Clinical or Referral files in CPRD GOLD, based on the Read codes detailed in Appendix 3, or in HES APC based on the ICD-10 codes in Appendix 4.
- 154,577 patients have above events before the end of the study period (31/12/2017).
- 138,403 patients have above events before the end of their UTS follow-up period.
- 132,972 patients are aged 18 years or less on the diagnosis event date.
- 7371 patients had a diagnosis on or before 31/12/2017 of life-limiting conditions, either in the Clinical or Referral files in CPRD GOLD based on the Read codes detailed in Appendix 1, or in HES APC based on the ICD-10 codes in Appendix 2 and were excluded.
- 0 children have deregistered from the CPRD on or before 01/04/2007, and were excluded.
- 1986 children are siblings of children in the case population (Group 1), based on having a link to the same mother in the Mother Baby Link, and were excluded.

123,615 patients meet all criteria.

105,392 patients were identified as mothers of children eligible for inclusion in Group 2 in the CPRD Mother Baby Link.

Control definition and matching (Group 2)

Of the 8950 cases, **8868** had one matched control in Group 2.

8732 patients were identified as mothers of children in this control group.

Eligible Control group (Group 3)

From the source population in CPRD GOLD:

- 10,598 patients had a diagnosis on or before 31/12/2017 of life-limiting conditions, either in the Clinical or Referral files in CPRD GOLD based on the Read codes detailed in Appendix 1, or in HES APC based on the ICD-10 codes in Appendix 2, and were excluded.
- 146,433 patients had a diagnosis on or before 31/12/2017 of chronic conditions that are not life-limiting, in the Clinical or Referral files in CPRD GOLD based on the Read codes detailed in Appendix 3, or in HES APC based on the ICD-10 codes in Appendix 4, and were excluded.
- 2 children have deregistered from the CPRD on or before 01/04/2007, and were excluded.
- 4769 children are siblings of children in the case population (Group 1), based on having a link to the same mother in the Mother Baby Link.

403,778 patients meet all criteria.

297,447 patients were identified as mothers of children eligible for inclusion in Group 3 in the CPRD Mother Baby Link.

Control definition and matching (Group 2)

Of the 8950 cases, 8930 had two matched controls in Group 3 and 8935 had at least one matched control. The total number of Group 3 controls was **17,865.**

17,477 patients were identified as mothers of children in this control group.

Criteria not applied by CPRD

None

Delivery population

A single dataset will be supplied containing all of the data for the children of interest and their mothers. This contains **70,213** patients.

Of these patients, all are eligible for linkage to ONS Death registration data, HES data and patient-level IMD. **70,169** patients are eligible for linkage to Mental Health Services Data set.

Table 2 Univariable Poisson models for mental health outcomes in mothers

	**Anxiety**			**Depression**			**Serious Mental Illness**			**Referral to secondary Mental Health services**		
	**IRR**	**95%CI**		**IRR**	**95%CI**		**IRR**	**95%CI**		**IRR**	**95%CI**	
**Child has no long term condition**	REF			REF			REF			REF		
**Child has a life-limiting condition**	1.19	*1.10*	*1.29*	1.27	*1.18*	*1.36*	1.85	*1.31*	*2.60*	1.72	*1.46*	*2.03*
**Child has a chronic condition**	1.26	*1.17*	*1.36*	1.27	*1.19*	*1.36*	1.47	*1.04*	*2.09*	1.39	*1.18*	*1.65*
**Mothers Age**	0.96	*0.95*	*0.96*	0.95	*0.95*	*0.95*	0.95	*0.93*	*0.97*	0.94	*0.93*	*0.95*
**Deprivation category**												
**1 (least deprived)**	REF			REF			REF			REF		
**2**	1.07	*0.97*	*1.18*	1.10	*1.01*	*1.20*	1.50	*0.90*	*2.51*	2.01	*1.56*	*2.59*
**3**	1.32	*1.20*	*1.46*	1.26	*1.15*	*1.37*	1.63	*0.98*	*2.72*	2.53	*1.98*	*2.25*
**4**	1.38	*1.25*	*1.52*	1.40	*1.28*	*1.53*	2.46	*1.54*	*3.94*	2.88	*2.26*	*3.67*
**5 (most deprived)**	1.52	*1.37*	*1.68*	1.64	*1.51*	*1.78*	2.81	*1.75*	*4.49*	3.24	*2.54*	*4.13*
**Ethnic Group**												
**White**	REF			REF			REF			REF		
**South Asian**	0.58	*0.49*	*0.69*	0.58	*0.51*	*0.67*	0.37	*0.14*	*0.99*	0.67	*0.47*	*0.95*
**Black**	0.39	*0.30*	*0.51*	0.57	*0.48*	*0.69*	0.66	*0.24*	*1.75*	0.55	*0.33*	*0.91*
**Chinese**	0.65	*0.38*	*1.12*	0.31	*0.17*	*0.58*	0.00	*0*		0.00	*0.00*	*.*
**Mixed**	0.97	*0.70*	*1.34*	1.04	*0.78*	*1.39*	1.75	*0.56*	*5.50*	0.69	*0.28*	*1.65*
**Other**	0.61	*0.45*	*0.82*	0.59	*0.45*	*0.77*	0.93	*0.30*	*2.92*	0.87	*0.49*	*1.54*
**Missing**	0.40	*0.33*	*0.48*	0.41	*0.36*	*0.48*	0.13	*0.03*	*0.54*	0.29	*0.18*	*0.45*
**Number of GP consults**	1.01	*1.01*	*1.01*	1.01	*1.01*	*1.01*	1.01	*1.01*	*1.01*	1.01	*1.01*	*1.01*
**Region**												
**North East**	2.46	*2.03*	*2.98*	1.87	*1.54*	*2.24*	1.28	*0.43*	*3.76*	0.98	*0.59*	*1.64*
**North West**	1.63	*1.44*	*1.83*	1.42	*1.28*	*1.57*	2.04	*1.20*	*3.48*	0.65	*0.49*	*0.88*
**Yorkshire & Humber**	1.26	*1.00*	*1.57*	1.09	*0.90*	*1.33*	0.28	*0.04*	*2.13*	1.30	*0.84*	*2.03*
**East Midlands**	2.12	*1.71*	*2.64*	2.01	*1.65*	*2.43*	3.55	*1.56*	*8.12*	1.68	*1.04*	*2.71*

**West Midlands**	1.51	*1.33*	*1.73*	1.37	*1.22*	*1.53*	1.56	*0.85*	*2.87*	1.42	*1.09*	*1.85*
**East of England**	1.08	*0.94*	*1.23*	0.94	*0.89*	*1.13*	1.04	*0.54*	*1.97*	0.59	*0.43*	*0.81*
**South West**	1.75	*1.54*	*1.98*	1.49	*1.34*	*1.66*	1.26	*0.68*	*2.35*	2.50	*1.98*	*3.14*
**South Central**	1.30	*1.14*	*1.48*	1.34	*1.20*	*1.50*	1.35	*0.75*	*2.45*	0.33	*0.22*	*0.49*
**London**	ref											
**South East Coast**	1.22	*1.07*	*1.39*	1.26	*1.13*	*1.41*	1.09	*0.58*	*2.03*	1.32	*1.03*	*1.70*
**Child sex**												
**Male**	Ref											
**Female**	0.95	*0.89*	*1.01*	1.00	*0.95*	*1.07*	0.91	*0.68*	*1.21*	1.03	*0.89*	*1.18*
**Baby birth year**	1.02	*1.02*	*1.03*	1.04	*1.03*	*1.04*	0.99	*0.97*	*1.02*	1.02	*1.01*	*1.03*

Fraser LK, *et al. Arch Dis Child* 2021; 106:987–993. doi: 10.1136/archdischild-2020-320655

Table 3 Univariable Models for Maternal Physical Health Outcomes

	**Obesity**		n=32,675	**Cardiovascular Disease**		n=35,122	**Hypertension**		n=33,904	**Type 2 Diabetes**		n=34,869	**Back Pain**		n=23,111
	**IRR**	**95%CI**		**IRR**	**95%CI**		**IRR**	**95%CI**		**IRR**	**95%CI**		**IRR**	**95%CI**	
**Child has no long term condition**	REF			REF			REF			REF			REF		
**Child has a life-limiting condition**	1.41	*1.28*	*1.55*	2.09	*1.54*	*2.83*	1.48	*1.3*	*1.66*	1.41	*1.16*	*1.71*	1.10	*1.03*	*1.18*
**Child has a chronic condition**	1.27	*1.15*	*1.39*	1.34	*0.96*	*1.86*	1.39	*1.24*	*1.56*	1.30	*1.08*	*1.58*	1.30	*1.22*	*1.38*
**Mothers Age**	0.96	*0.95*	*0.97*	1.10	*1.08*	*1.12*	1.07	*1.06*	*1.07*	1.05	*1.04*	*1.06*	0.98	*0.98*	*0.99*
**Deprivation category**															
**1 (least deprived)**	REF			REF			REF			REF			REF		
**2**	1.75	*1.52*	*2.02*	1.22	*0.74*	*2.02*	1.03	*0.88*	*1.21*	0.97	*0.72*	*1.30*	1.13	*1.05*	*1.23*
**3**	2.14	*1.86*	*2.46*	1.95	*1.23*	*3.09*	1.30	*1.12*	*1.50*	1.63	*1.25*	*2.12*	1.20	*1.10*	*1.30*
**4**	2.69	*2.35*	*3.07*	2.85	*1.86*	*4.38*	1.51	*1.31*	*1.74*	2.07	*1.61*	*2.66*	1.33	*1.23*	*1.44*
**5 (most deprived)**	3.38	*2.96*	*3.85*	2.84	*1.83*	*4.39*	1.48	*1.28*	*1.72*	2.47	*1.93*	*3.17*	1.47	*1.36*	*1.59*
**Ethnic Group**															
**White**	REF			REF			REF			REF			REF		
**South Asian**	1.28	*1.10*	*1.50*	1.46	*0.90*	*2.37*	1.37	*1.13*	*1.65*	3.35	*2.68*	*4.18*	1.37	*1.24*	*1.52*
**Black**	1.52	*1.25*	*1.84*	1.01	*0.47*	*2.14*	2.25	*1.83*	*2.76*	1.84	*1.27*	*2.66*	1.26	*1.10*	*1.44*
**Chinese**	0.09	*0.01*	*0.62*	0.00	*0.00*	.	1.05	*0.50*	*2.20*	0.44	*0.06*	*3.14*	0.56	*0.35*	*0.91*
**Mixed**	0.87	*0.55*	*1.38*	0.00	*0.00*	.	1.27	*0.79*	*2.05*	0.66	*0.21*	*2.05*	1.13	*0.87*	*1.47*
**Other**	0.78	*0.55*	*1.12*	1.11	*0.41*	*2.98*	0.92	*0.61*	*1.39*	1.29	*0.71*	*2.34*	1.03	*0.84*	*1.25*
**Missing**	0.36	*0.28*	*0.45*	0.24	*0.09*	*0.65*	1.11	*0.92*	*1.33*	0.61	*0.40*	*0.92*	0.59	*0.52*	*0.66*
**Number of GP consults**	1.01	*1.01*	*1.01*	1.01	*1.01*	*1.01*	1.01	*1.01*	*1.01*	1.01	*1.01*	*1.01*	1.01	*1.01*	*1.01*
**Smoking**				1.46	*1.12*	*1.89*	1.19	*1.09*	*1.32*						
**Region**															
**North East**	**1.21**	0.93	1.57	1.46	*0.53*	*4.07*	1.21	*0.85*	*1.73*	1.30	0.78	2.19	1.06	0.89	1.27
**North West**	**1.03**	0.88	1.19	1.12	*0.30*	*4.18*	1.25	*1.04*	*1.51*	1.12	0.84	1.51	1.06	0.97	1.16
**Yorkshire & Humber**	**0.98**	0.74	1.29	2.92	*0.88*	*9.76*	1.10	*0.78*	*1.54*	1.79	1.13	2.85	0.98	0.82	1.16
**East Midlands**	**2.31**	1.82	2.93	1.16	*0.40*	*3.33*	2.05	*1.49*	*2.83*	2.39	1.46	3.93	1.15	0.95	1.40
**West Midlands**	**1.28**	1.09	1.49	1.94	*0.68*	*5.49*	1.16	*0.95*	*1.41*	1.03	0.75	1.41	1.00	0.90	1.11
**East of England**	**1.07**	0.91	1.27	1.12	*0.39*	*3.22*	1.38	*1.14*	*1.67*	1.09	0.79	1.50	0.96	0.87	106

**South West**	**1.24**	1.06	1.44	1.12	*0.38*	*3.32*	1.02	*0.83*	*1.25*	1.07	0.77	1.47	0.99	0.90	1.12
**South Central**	**1.06**	0.89	1.25	0.92	*0.31*	*2.70*	1.15	*0.94*	*1.41*	1.08	0.78	1.50	1.01	0.84	1.02
**London**	**REf**														
**South East Coast**	**0.87**	0.74	1.03	1.29	*0.44*	*3.79*	1.23	*1.01*	*1.50*	0.89	0.62	1.26	0.92	0.84	1.02
**Child sex**															
**Male**															
**Female**	**0.99**	0.91	1.07	0.84	*0.64*	*1.09*	0.96	*0.87*	*1.06*	0.85	0.72	1.00	0.92	0.87	0.97
**Baby birth year**	**1.02**	1.02	1.03	0.92	*0.90*	*10.94*	0.94	*0.93*	*0.95*	0.95	0.94	0.96	1.01	1.00	1.02

Fraser LK, *et al. Arch Dis Child* 2021; 106:987–993. doi: 10.1136/archdischild-2020-320655
